# Antibodies against SARS-CoV-2 Suggestive of Single Events of Spillover to Cattle, Germany

**DOI:** 10.3201/eid2809.220125

**Published:** 2022-09

**Authors:** Kerstin Wernike, Jens Böttcher, Silke Amelung, Kerstin Albrecht, Tanja Gärtner, Karsten Donat, Martin Beer

**Affiliations:** Friedrich-Loeffler-Institut, Greifswald–Insel Riems, Germany (K. Wernike, M. Beer);; Bavarian Animal Health Service, Poing, Germany (J. Böttcher);; LUFA Nord-West, Oldenburg, Germany (S. Amelung);; State Institute for Consumer Protection of Saxony-Anhalt, Stendal, Germany (K. Albrecht);; Thuringian Animal Diseases Fund, Animal Health Service, Jena, Germany (T. Gärtner, K. Donat)

**Keywords:** COVID-19, 2019 novel coronavirus disease, coronavirus disease, severe acute respiratory syndrome coronavirus 2, SARS-CoV-2, viruses, respiratory infections, zoonoses, animal, reservoir, cattle, ruminants, livestock, serology, epidemiology, Germany

## Abstract

Human infection with SARS-CoV-2 poses a risk for transmission to animals. To characterize the risk for cattle, we serologically investigated 1,000 samples collected from cattle in Germany in late 2021. Eleven antibody-positive samples indicated that cattle may be occasionally infected by contact with SARS-CoV-2–positive keepers, but we found no indication of further spread.

Since its first detection at the end of 2019, SARS-CoV-2, which induces COVID-19 in humans, very rapidly spread around the world, causing a massive global pandemic that resulted in >5 million deaths in <2 years of virus circulation (*1*). Since the beginning of the pandemic, researchers have discussed the role of livestock and wildlife species at the human–animal interface, with a special focus on the identification of susceptible species and potential intermediate or reservoir hosts.

Under experimental conditions, various animal species could be infected with SARS-CoV-2, including nonhuman primates, felids, canids, mustelids, white-tailed deer, and several species of *Cricetidae* rodents; poultry or swine were not susceptible (*2*). For domestic ruminants such as cattle, sheep, or goats, susceptibility after experimental inoculation was low; only a small proportion of animals could be infected without animal-to-animal transmission (*3*–*5*). Furthermore, 26 cattle exposed in the field to SARS-CoV-2 by contact with their infected keepers tested negative by reverse transcription PCR (*6*). However, given the very short time at which cattle test positive by reverse transcription PCR after experimental infection (1–2 days) (*3*,*7*), serologic screening could be more beneficial for identifying previously infected animals and estimating the rate of spillover infections in the field.

We analyzed 1,000 serum or plasma samples from cattle at 83 farms in 4 federal states in Germany (Bavaria, Lower Saxony, Saxony-Anhalt, and Thuringia). Because the samples represented superfluous material from routine diagnostic submissions by the responsible veterinarians in the context of the health monitoring of the respective cattle farm, no permissions were needed to collect these specimens. Sampling dates were autumn 2021 and early winter 2021–22, during a massive wave of infections in the human population driven by the SARS-CoV-2 Delta variant of concern. We analyzed 2–20 randomly selected serum or plasma samples per farm (Figure). Farm 31 was sampled twice; between farm samplings, the animal owner was quarantined. We do not know whether this quarantine resulted from contact with an infected person or whether the owner himself tested SARS-CoV-2 positive. All bovine samples were tested by a receptor-binding domain (RBD)–based multispecies ELISA (diagnostic sensitivity 98.31% and specificity 100%) performed as described previously (*8*). Initial test validation and an experimental SARS-CoV-2 infection study in cattle have demonstrated that the ELISA does not cross-react with the bovine coronavirus (BCoV) (*3*,*8*). We investigated an additional 100 cattle control samples randomly collected across Germany in 2016, and all tested negative.

**Figure:**
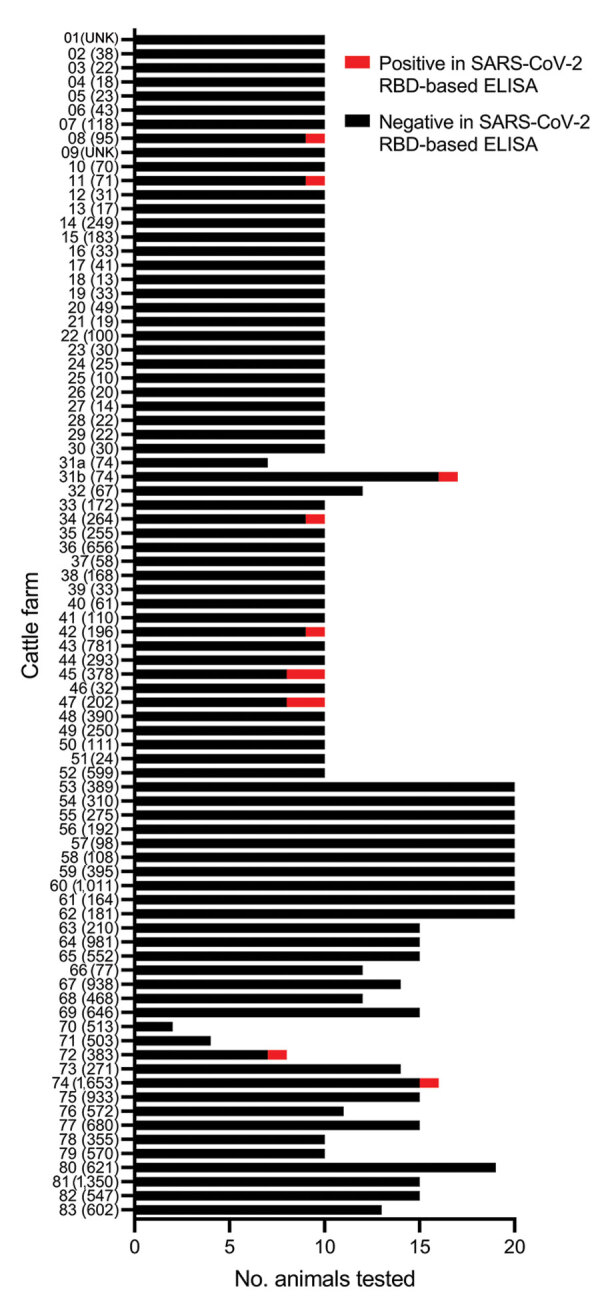
Number of cattle per farm tested for antibodies against SARS-CoV-2, Germany, 2021. Numbers in parentheses indicate herd size. Black bar sections indicate samples with negative reaction in the RBD-based ELISA; red bar sections indicate positive samples. Farm 31 was sampled twice (indicated as 31a and 31b), before and after animal owner quarantine. RBD, receptor-binding domain; UNK, unknown.

Of the cattle sampled in 2021, eleven animals from 9 farms tested positive by the RBD ELISA; among them was 1 animal on farm 31, sampled after the owner was quarantined (Figure). Positive ELISA results for all but 1 sample (farm 8) could be confirmed by an indirect immunofluorescence assay that used Vero cells infected with the SARS-CoV-2 strain 2019_nCoV Muc-IMB-1 (multiplicity of infection of 0.1) as antigen matrix (*3*). Titers ranged from 1:8 through 1:512, and the highest titer was from the seropositive animal on farm 31 (Table). To further confirm the reactivity toward SARS-CoV-2, we additionally tested the 11 samples that reacted positive in the RBD-ELISA by using a surrogate virus neutralization test (cPass SARS-CoV-2 Surrogate Virus Neutralization Test [sVNT] Kit; GenScript, https://www.genscript.com). This test enables detection of neutralizing antibodies by mimicking the interaction between SARS-CoV-2 and host cell membrane receptor protein ACE2; it is reportedly highly specific but only moderately sensitive for animal samples because it does not detect low antibody titers (*9*). sVNT produced positive results for 4 cattle (farms 11, 31, 47, and 74; Table).

**Table:** Results of samples that tested positive by a multispecies SARS-CoV-2 RBD-based ELISA, Germany, 2021*

Cattle farm/animal number (federal state)	RBD-ELISA, corrected OD	Indirect IFA, titer	sVNT, % inhibition
8/1 (Bavaria)	**0.35 **	<1:8	6.1
11/1 (Bavaria)	**0.70 **	**1:32 **	**36.4 **
31/1 (Bavaria)	**1.00 **	**1:512 **	**57.8 **
34/1 (Lower Saxony)	**0.50 **	**1:32 **	11.7
42/1 (Lower Saxony)	**0.65 **	**1:16 **	5.5
45/1 (Lower Saxony)	**0.67 **	**1:8 **	10.6
45/2 (Lower Saxony)	**0.33 **	**1:16 **	9.0
47/1 (Lower Saxony)	**0.48 **	**1:8 **	**37.1 **
47/2 (Lower Saxony)	**0.67 **	**1:8 **	0.6
72/1 (Thuringia)	**0.52 **	**1:16 **	4.7
74/1 (Thuringia)	**0.76 **	**1:32 **	**54.2 **

*Boldface indicates positive results. IFA, immunofluorescence assay; OD, optical density; RBD, receptor-binding domain; sVNT, surrogate virus neutralization test (cPass SARS-CoV-2 Surrogate Virus Neutralization Test Kit; GenScript, https://www.genscript.com; cutoff >30% positive and <30% negative).

Our findings of a low number of individual seropositive cattle on several farms demonstrate that cattle might be occasionally infected and seroconvert after contact with infected humans. However, in keeping with experimental infection studies (*3*), intraspecies transmission seems likewise to not occur in the field. Nevertheless, cattle farms should be included in future monitoring programs, especially because another coronavirus (i.e., BCoV) is highly prevalent in the cattle population and a BCoV infection did not prevent a SARS-CoV-2 infection in a previous study (*3*). Furthermore, we do not know the susceptibility of animal hosts for the Omicron variant. Double infections of individual animals could potentially lead to recombination between both viruses, a phenomenon described for other coronaviruses (*10*). Although emergence is highly unlikely because of the low susceptibility of cattle for SARS-CoV-2, a conceivable chimera between SARS-CoV-2 and BCoV could represent an additional threat. Hence, ruminants should be included in outbreak investigations, and regular screenings should be performed to exclude any spread of new variants in the livestock population.

This article was originally a preprint (https://www.biorxiv.org/content/10.1101/2022.01.17.476608v1).
